# Size-Controllable Enzymatic Synthesis of Short Hairpin RNA Nanoparticles by Controlling the Rate of RNA Polymerization

**DOI:** 10.3390/polym10060589

**Published:** 2018-05-28

**Authors:** Hyejin Kim, Dajeong Kim, Jaepil Jeong, Hyunsu Jeon, Jong Bum Lee

**Affiliations:** Department of Chemical Engineering, University of Seoul, Seoul 02504, Korea; hyejinkim@uos.ac.kr or ajin2010@uos.ac.kr (H.K.); djkimuos@gmail.com (D.K.); jpjeong@uos.ac.kr (J.J.); hyunsu0057@uos.ac.kr (H.J.)

**Keywords:** RNA nanotechnology, RNA therapeutics, short hairpin RNA nanoparticles, enzymatic self-assembly

## Abstract

Thanks to a wide range of biological functions of RNA, and advancements in nanotechnology, RNA nanotechnology has developed in multiple ways for RNA-based therapeutics. In particular, among RNA engineering techniques, enzymatic self-assembly of RNA structures has gained great attention for its high packing density of RNA, with a low cost and one-pot synthetic process. However, manipulation of the overall size of particles, especially a reduction in size, has not been studied in depth. Here, we reported the enzymatic self-assembly of short hairpin RNA particles for the downregulation of target genes, and a rational approach to the manipulation of the resultant particle size. This is the first report of the size-controllable enzymatic self-assembly of short hairpin RNA nanoparticles. While keeping all the benefits of an enzymatic approach, the overall size of the RNA particles was controlled on a scale of 2 μm to 100 nm, falling within the therapeutically applicable size range.

## 1. Introduction

RNA nanotechnology has developed enormously by taking advantage of the intrinsic properties of RNA, and advancements in nanotechnology [1,2,3]. The major advantages of RNA encompass its programmability, and its various biological functions. The programmability of RNA originates from its simple molecular structure, comprised of four monomers—adenine, uracil, guanine, and cytosine. Each monomer is involved in the enzymatic process of RNA polymerization, and the resulting polymeric strand of RNA can be hybridized in a sequence-specific manner by Watson–Crick base pairing [4,5]. In addition, the resulting strand of RNA can be folded into complicated higher-order structures with various interactions, and other related structures, such as stem-loop structures, sticky ends, and loop–loop interactions [6,7]. Furthermore, RNA has a wide range of biological functions, and takes various forms dependent on each one. Messenger RNAs, transfer RNAs, ribosomal RNAs, ribozymes, riboswitches, small interfering RNAs, microRNAs, and small nuclear RNAs are examples of various types of RNAs with distinct functions.

To fully exploit RNA, RNA nanotechnology has been developed in many ways. For the synthesis of functional RNA structures, a simple hybridization approach was used for the generation of a polyhedral structure, while a crossover technique and RNA architectonics were used for the generation of more complex RNA structures [8,9,10,11]. These RNA-based nanostructures were applied to a range of therapeutic applications, including the targeting of specific tumor cells in vivo, immunotherapy, and the delivery of chemotherapy drugs [2,12,13]. Among the range of RNA engineering approaches, the enzymatic replication and simultaneous self-assembly of RNA has gained great attention due to its potential of synthesizing various types of self-assembled structures for designated biological functions, ranging from nanometer-sized particles to centimeter-scale membranes [14,15,16]. An enzymatic approach has the benefit of achieving high packing density at a lower cost when compared with other approaches, and of enabling the one-step fabrication of artificial RNA-based structures of various sizes. Taking advantage of the benefits of building RNA structures through an enzymatic process, RNA-based structures have been widely used for therapeutic purposes such as non-viral protein expression, anti-proliferation of tumors, and treatment of choroidal neovascularization [15,17,18]. 

Here, we report the size-controllable assembly of short hairpin RNA (shRNA) particles via an enzymatic approach. While the synthesis of size-controllable RNA nanoparticles was possible using complementary rolling circle transcription with two types of circular DNA templates [19,20], this represents the first report of the size-controllable enzymatic self-assembly of shRNA nanoparticles from single type circular DNA template. While keeping all the benefits of an enzymatic approach, the overall size of the RNA particles was controlled on a scale of 2 μm to 100 nm, falling within the therapeutically applicable size range [21,22].

## 2. Materials and Methods

### 2.1. Materials

All oligonucleotides were purchased from Integrated DNA Technologies (IDT, Skokie, IL, USA). T4 ligase was obtained from Promega (Madison, WI, USA). T7 RNA polymerase and ribonucleotide triphosphates (rNTPs) were purchased from New England BioLabs (NEB, Ipswich, MA, USA). Zeba^TM^ spin desalting columns were purchased from Thermo Fisher Scientific (Waltham, MA, USA). Cyanine 5-UTP was purchased from Enzo Life Sciences (Farmingdale, NY, USA). Mica and Lacey Formvar/carbon-coated copper grids (01883-F) were obtained from Ted Pella (Redding, CA, USA). Dulbecco’s modified Eagle’s medium (DMEM), penicillin/streptomycin (P/S), fetal bovine serum (FBS), and Dulbecco’s phosphate buffered saline (DPBS) were purchased from Gibco (Waltham, MA, USA). The Stemfect^TM^ RNA Transfection Kit was purchased from Stemgent (Houston, TX, USA). CelLytic M and MgCl_2_ solution were purchased from Sigma-Aldrich (Saint Louis, MI, USA). A Cell Counting Kit-8 (CCK-8) was obtained from Dojindo Laboratories (Kumamoto, Japan). Tris-HCl buffer was purchased from Invitrogen (Waltham, MA, USA).

### 2.2. Circularization of Anti-GFP shRNA Encoding Single-Stranded DNA

A 92-base-long phosphorylated linear DNA for anti-GFP shRNA was mixed with a 22-base-long primer DNA for T7 RNA polymerase at a final concentration of 3.0 μM in nuclease-free water (the sequences of oligonucleotides are shown in Table 1). The mixture was heated for 2 min at 95 °C, and gradually cooled down to 25 °C for 1 h using a thermal cycler (T100^TM^ Thermal Cycler, Bio-Rad, Hercules, CA, USA). T4 ligase (0.06 units/µL) was then introduced to the mixture with ligase buffer (30 mM Tris-HCl, 10 mM MgCl_2_, 10 mM DTT, and 1 mM ATP). The solution was incubated overnight at room temperature to ligate the nick in the circularized DNA.

### 2.3. Synthesis of shRNA Particles

The circular DNA at final concentrations of 0.03 μM, 0.1 μM, or 0.3 μM were mixed with 8 mM ribonucleotide solution mix, reaction buffer (80 mM Tris, 40 mM NaCl, 12 mM MgCl_2_, 4 mM spermidine, and 20 mM dithiothreitol; pH 7.8), and 5 units/μL T7 RNA polymerase. For the rolling circle transcription (RCT) reaction, the reaction solution was incubated for 20 h at 37 °C. The final reaction solution was briefly sonicated, before the shRNA particles were purified with a Zeba^TM^ Desalting Column, following the manufacturer’s protocol. For the synthesis of cy5-labeled shRNA-NPs, cy5-UTP (final concentration of 20 μM) was added to the RCT reaction mixture at the beginning of the incubation process. To remove unincorporated cy5-UTP, the shRNA-NPs were purified with a Zeba^TM^ Desalting Column after the RCT reaction. 

### 2.4. Characterization

A field emission scanning electron microscope (FE-SEM) (S-5000H, Hitachi, Tokyo, Japan) and an atomic force microscope (AFM) (Park NX10, Park Systems, Suwon, South Korea) were used to obtain high-resolution digital images of the shRNA particles. The shRNA particles for SEM imaging were deposited onto a silicon wafer, and coated with Pt after being dried. For AFM imaging, 10 μL of the reaction mixture was diluted in nuclease-free water containing 5 mM Tris-HCl and 5 mM MgCl_2_. After incubating the mixture at 4 °C for 30 min, 50 μL of the mixture was deposited onto freshly cleaved mica surface, and further incubated at 4 °C for 30 min. Following the incubation, the mica surface was rinsed with deionized water to remove salts, and nitrogen gas was then sprayed onto the surface for three to five seconds to remove the remaining solution. The samples were scanned in non-contact mode with NC-NCH tips (Park Systems). Nanoparticle tracking analysis was carried out with Nanoparticle Tracking Analysis (NanoSight NS300, Malvern, Worcestershire, UK). Transmission electron microscopy (TEM) (JEM-2100F, JEOL, Tokyo, Japan) was employed to characterize the shRNA-NPs, operating at an accelerated voltage of 200 kV, before TEM-based energy dispersion X-ray (EDX) was used to analyze the chemical compositions of the shRNA-NPs. For the preparation of samples, the shRNA-NPs were deposited onto Lacey Formvar/carbon-coated copper grids, and then air-dried at room temperature.

### 2.5. Intracellular Uptake Analysis

HeLa cells were grown in DMEM, supplemented with 10% FBS, 100 units/mL penicillin, 100 μg/mL streptomycin, and 1% Antibiotic-Antimycotic, at 37 °C in a humidified atmosphere, supplemented with 5% CO_2_. The cells were passaged routinely to maintain exponential growth. One day prior to transfection (~90% confluence), the cells were trypsinized, diluted with fresh medium, and transferred to 24-well plates (50,000 cells per well). The cy5-labeled shRNA-NPs were covered with the delivery carrier, a Stemfect^TM^ RNA Transfection Kit, following the manufacturer’s instructions. Specifically, the cy5-labeled shRNA-NPs were mixed with the Stemfect^TM^ RNA Transfection reagent (RNA:reagent = 1:3 *w*/*v*) in PBS solution, and incubated for 10 min at room temperature. After diluting the shRNA-NPs/reagent solution with media, the samples were treated to cells for 4 h at a concentration of 2.5 μg/mL at 37 °C. After further incubation for 12 h in fresh serum-containing media, the cells were detached from the plates by treatment with trypsin-EDTA solution, and washed three times with PBS. The cells were analyzed by NucleoCounter (NC-3000, Chemometec, Allerod, Denmark). The data were analyzed using the FlowJo software.

### 2.6. In Vitro Gene Knockdown Analysis

HeLa-GFP cells were transferred to 96-well plates (7000 cells per well). The shRNA-NPs were covered with the transfection reagent from the Stemfect^TM^ RNA Transfection Kit, prior to transfection according to the manufacturer’s instructions. Then, the cells were treated with various concentrations of the covered shRNA-NPs, ranging from 0.1 to 2.5 μg/mL. After 24 h of treatment, cells were washed with DPBS, and lysed with CelLytic M. The green fluorescence from each well containing the lysed cells was detected by a microplate reader (Synergy HT, BioTek, Winooski, VT, USA), and then normalized with the green fluorescence from the well containing untreated HeLa-GFP cell lysates, to obtain relative GFP expression. Cell viabilities were assessed with CCK-8 according to the manufacturer’s instructions. 

### 2.7. Statistical Analysis 

Data in this study were represented as mean values of independent measurements (*n* = 4). Error bars indicated mean standard deviations of each experiment. Statistical analysis was performed with a Student’s *t*-test. Statistical significance was assigned for *p* < 0.05 (95% confidence level).

## 3. Results and Discussion

**Figure 1:**
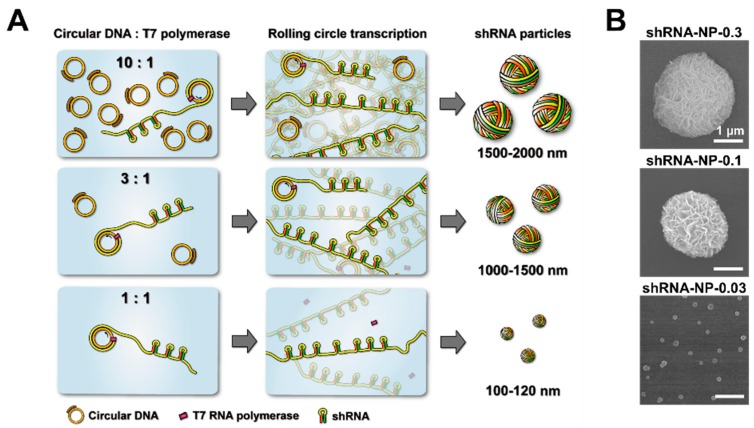
(**A**) Schematic illustration outlining the size-controlled synthesis of short hairpin RNA (shRNA) particles. By controlling concentrations of circular DNA in the rolling circle transcription (RCT) reaction at the same unit concentration of T7 RNA polymerase, the sizes of resulting RCT-mediated self-assembled shRNA particles could be controlled; (**B**) Scanning electron microscopy (SEM) images of shRNA particles showing their different sizes.

For the synthesis of shRNA particles, circularized template DNA was first prepared as described in previous reports [23,24]. Then, to synthesize size-controlled shRNA particles via rolling circle transcription, various concentrations ranging from 0.03 to 3.0 μM were mixed with T7 RNA polymerase, and other reaction components for polymerizing RNA strands (Figure 1). In the previous study, we reported that the concentration ratio of circular DNA to polymerase played an important role in controlling the size of RNA nanoparticles [19,20]. That was for complementary rolling circle transcription which involved two types of circular DNAs that were complementary to each other. Here, we report that the same logic could be applied to controlling the sizes of shRNA particles via rolling circle transcription that only involved one type of circular DNA. Downsizing of the RNA nanoparticles was achieved not only by increasing the concentration of polymerase, but also by decreasing the concentration of circular DNA in the RCT reaction. This was coherent with previous findings that the ratio of circular DNA to polymerase was the main factor in manipulating the sizes of final self-assembled products. Interestingly, however, manipulation of the concentration ratio of circular DNA to polymerase, by increasing concentration of RNA polymerase with one type of circular DNA, did not result in reducing the size of the particles (Figure S1).

**Figure 2:**
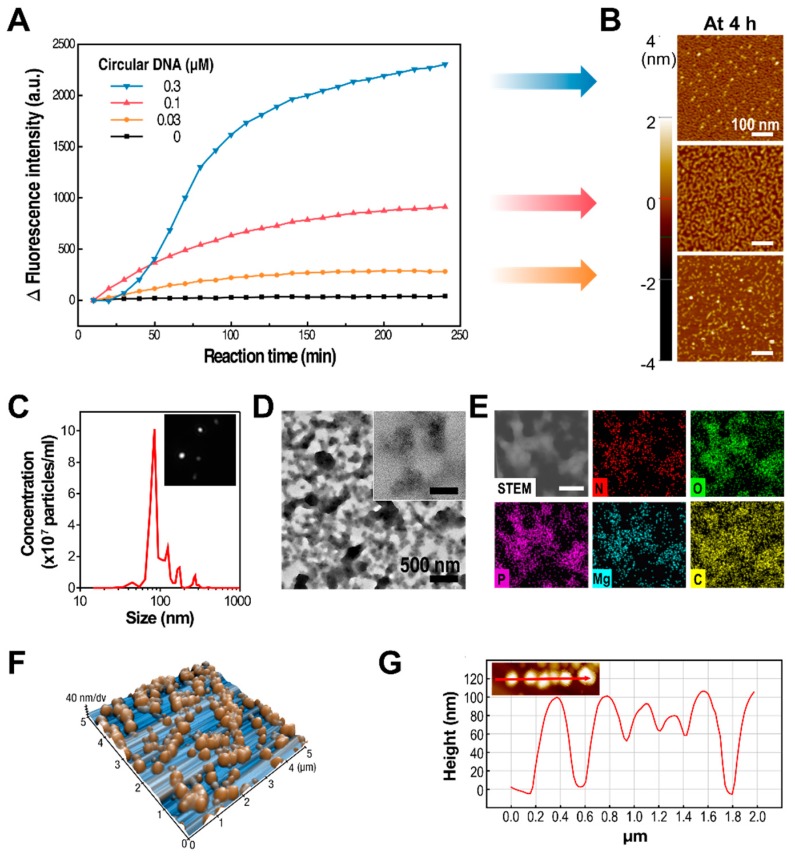
(**A**,**B**) Real-time analysis of RNA generation during the RCT reaction with various concentrations of circular DNAs (**A**), and the corresponding atomic force microscopy (AFM) images of the RCT products at 4 h (**B**). (**C**) Nanoparticle tracking analysis of shRNA nanoparticles (shRNA-NPs), revealing their size distribution and their concentrations. Inset image was captured from Supplementary Video 1, showing the scattered lights from nanoparticles exhibiting Brownian motion in solution. (**D**,**E**) Transmission electron microscopy (TEM) image and high-resolution TEM image (inset) of shRNA-NPs (**D**, inset scale bar: 100 nm), and TEM-based energy dispersion X-ray (EDX) mapping result (**E**, inset scale bar: 400 nm), showing the atomic compositions of the NPs. (**F**,**G**) AFM image of shRNA-NPs reconstructed in 3D (**F**), and cross-sectional analysis of the AFM image along the red line shown in the inset image (**G**).

To better understand the dependence of the synthetic process and its resulting products on the concentration of the circularized DNA template in the RCT reaction, RNA amplification under different synthetic conditions was observed in real time with RT-PCR. For the initial four hours of the RCT reaction, increasing the concentration of the circularized DNA template resulted in a higher number of RNA strands synthesized by T7 RNA polymerase (Figure 2A). Interestingly, changes in fluorescence intensity were directly proportional to the concentration of circular DNAs, even though the concentrations of the monomers and the enzymes were kept the same. This indicated that the amount of RNA generated by the RCT reaction could be controlled by changing the concentration of circular DNAs. This is logical when considering that polymerases are likely to work on fully constructed circular DNAs, rather than those already being used by the polymerase to transcribe RNA strands. Correspondingly, the RCT products from various concentrations of circular DNAs at 4 h of reaction were closely examined by atomic force microscopy (AFM, Figure 2B). Coherently with the real-time PCR result, the amount of synthesized RNA was greater at higher concentrations of template DNA. In addition, the level of entanglement of RNA strands was also higher at higher concentrations of template DNA.

In order to test the therapeutic efficacy of shRNA particles, we chose shRNA nanoparticles (shRNA-NPs) synthesized with 0.03 μM of circular DNA to further characterize their nature. The self-assembled shRNA-NPs had spherical structures that were 100 nm in diameter, revealed by scanning electron microscopy (SEM, Figure 1B), and nanoparticle tracking analysis (NTA, Figure 2C). Also, NTA results indicated a narrow size distribution of the nanoparticles, indicating that the shRNA-NPs had a favorable size allowing the cellular internalization of the shRNAs to be released from the nanoparticles for the regulation of target genes. Also, transmission electron microscopy (TEM) images revealed that the nanoparticles were homogeneous from their core to their outermost region, indicating that shRNAs were present throughout the particles (Figure 2D). The chemical composition of the nanoparticles included phosphorus and nitrogen, which indicated the existence of the phosphate backbones and nucleobases of nucleic acids in the structure (Figure 2E). Each of the atomic contents was evenly distributed according to TEM-based mapping, showing a uniform distribution of nucleic acids in the nanoparticles.

While images taken from SEM and TEM provide only two-dimensional information about the nanoparticles, the height of the nanoparticles was measured with atomic force microscopy, which is known for excellent *z*-axial resolution, so as to determine the full three-dimensional structure (Figure 2F). The overall height of the nanoparticles was about 100 nm, which supports the shRNA-NPs having fully spherical structures, along with previous data on the nanoparticles (Figure 2G). This was further supported by the NTA, which tracked individual nanoparticles exhibiting Brownian motion (Figure 2C, Supplementary Video S1). The hydrodynamic diameter of the nanoparticles was also measured to be approximately 100 nm, which indicated that the nanoparticles stayed compact even in hydrated conditions. 

**Figure 3:**
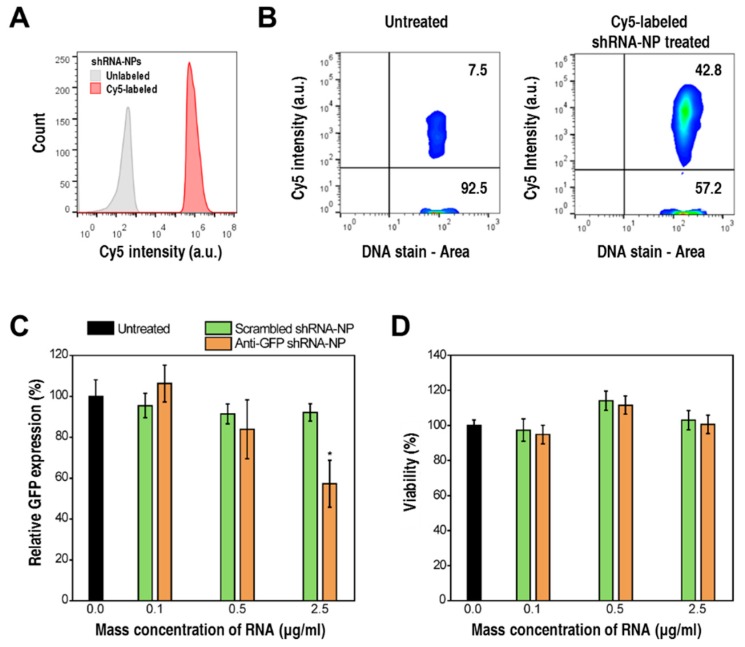
(**A**) Cytometry analysis of unlabeled (gray) and cy5-labeled shRNA-NPs, showing that fluorescent labeling of shRNA-NPs was successfully carried out. (**B**) Cellular-uptake analysis of HeLa cells treated with unlabeled or cy5-labeled shRNA-NPs. (**C**,**D**) Relative GFP expression (**C**) and cell viabilities (**D**) of HeLa-GFP cells treated with various concentrations of negative control (green), anti-GFP shRNA-NPs (orange), or left untreated (black) at 24 h after the treatment (* *p* < 0.05).

To evaluate cellular internalization readily by cytometry, the shRNA-NPs were enzymatically engineered with fluorescence-emitting modified nucleotides, cyanine 5-UTPs (cy5-UTP). By introducing cy5-UTPs into the RCT reaction, T7 RNA polymerase incorporated these molecules into synthesized RNA strands. Thus, the resulting self-assembled shRNA-NPs emitted red fluorescence, resulting in an increased red fluorescence signal when analyzed by image cytometry (Figure 3A). Accordingly, cytometry analysis was also carried out for HeLa cells treated with cy5-labeled shRNA-NPs, for the evaluation of cellular internalization. As shown in Figure 3B, there was a 6-fold increase in the number of cells showing a strong cy5 signal, which indicated a successful cellular uptake of the shRNA-NPs.

To test the gene-silencing activities of shRNA-NPs, anti-GFP shRNA-NPs were treated to HeLa cells stably expressing GFP (HeLa-GFP). The expression levels of GFP went down by 50% when treated with 2.5 μg RNA per ml (Figure 3C), while the treatment with shRNA-NPs did not cause any significant cytotoxic effects (Figure 3D). Furthermore, non-targeting shRNA-NPs showed negligible effects on the level of GFP expression, which proved a target-specific gene-regulation effect of shRNA-NPs, without causing any adverse side effects. 

## 4. Conclusions

In summary, we developed size-controllable synthesis of short hairpin RNA nanoparticles via an enzymatic approach. By controlling the concentrations of template circular DNAs while maintaining the same concentration of RNA polymerase in the RCT reaction, the sizes of the shRNA particles could be reduced from 2 μm to 100 nm. The resulting shRNA-NPs were fully characterized, and controlling their sizes was possible through controlling the level of entanglement of RNA strands by changing concentrations of template DNA in the RCT reaction. In addition, the shRNA-NPs had a favorable size for therapeutic applications. Through such a development in RNA engineering, we envision that we can step forward into the real-world applications of RNA therapeutics.

## Figures and Tables

**Table 1:** DNA sequences for synthesizing anti-GFP, and negative control short hairpin RNA nanoparticles (shRNA-NPs). The complementary DNA sequence for the promoter region of T7 RNA polymerase is shown in blue, and the primer for T7 RNA polymerase binds to the blue region to form the promoter region of T7 RNA polymerase.

DNA Strands	Length (nt)	Sequence
Linear DNA for anti-GFP shRNA-NPs	92	5’-phosphate-ATA GTG AGT CGT ATT AAC GTA CCA ACA AAA CTT CAG GGT CAG CTT GCT TAC TTG AAG CAA GCT GAC CCT GAA GTT TTT AGA GGC ATA TCC CT-3’
Linear DNA for non-targeting shRNA-NPs	92	5’-Phosphate-ATA GTG AGT CGT ATT AAC GTA CCA ACA ACT TAC GCT GAG TAC TTC GAT TAC TTG AAT CGA AGT ACT CAG CGT AAG TTT AGA GGC ATA TCC CT-3’
Primer for T7 RNA polymerase	22	5’-TAA TAC GAC TCA CTA TAG GGA T-3’

## Supplementary Materials

The supplementary materials are available online at http://www.mdpi.com/2073-4360/10/6/589/s1.
