# Supplementary material for: Size-Controllable Enzymatic Synthesis of Short Hairpin RNA Nanoparticles by Controlling the Rate of RNA Polymerization
Source: Polymers (Basel). 2018 May 28;10(6):589. doi: 10.3390/polym10060589 (PMC6403749; doi:10.3390/polym10060589)
Supplement: Supplementary file 1 [file polymers-10-00589-s001.zip › Supplementary Information_Size-controllable enzymatic synthesis of short hairpin RNA nanoparticles by controlling the rate of RNA polymerization.docx]

**Supplementary Information**

Size-controllable Enzymatic Synthesis of Short Hairpin RNA Nanoparticles by Controlling the Rate of RNA Polymerization

*Hyejin Kim, Dajeong Kim, Jaepil Jeong, Hyunsu Jeon, Jong Bum Lee^*^*

Department of Chemical Engineering, University of Seoul, Seoul 02504, Republic of Korea

*Fax: +82-2-6490-2364; Tel: +82-2-6490-2372

Correspondence and requests for materials should be addressed to J.B.L. (e-mail: jblee@uos.ac.kr).


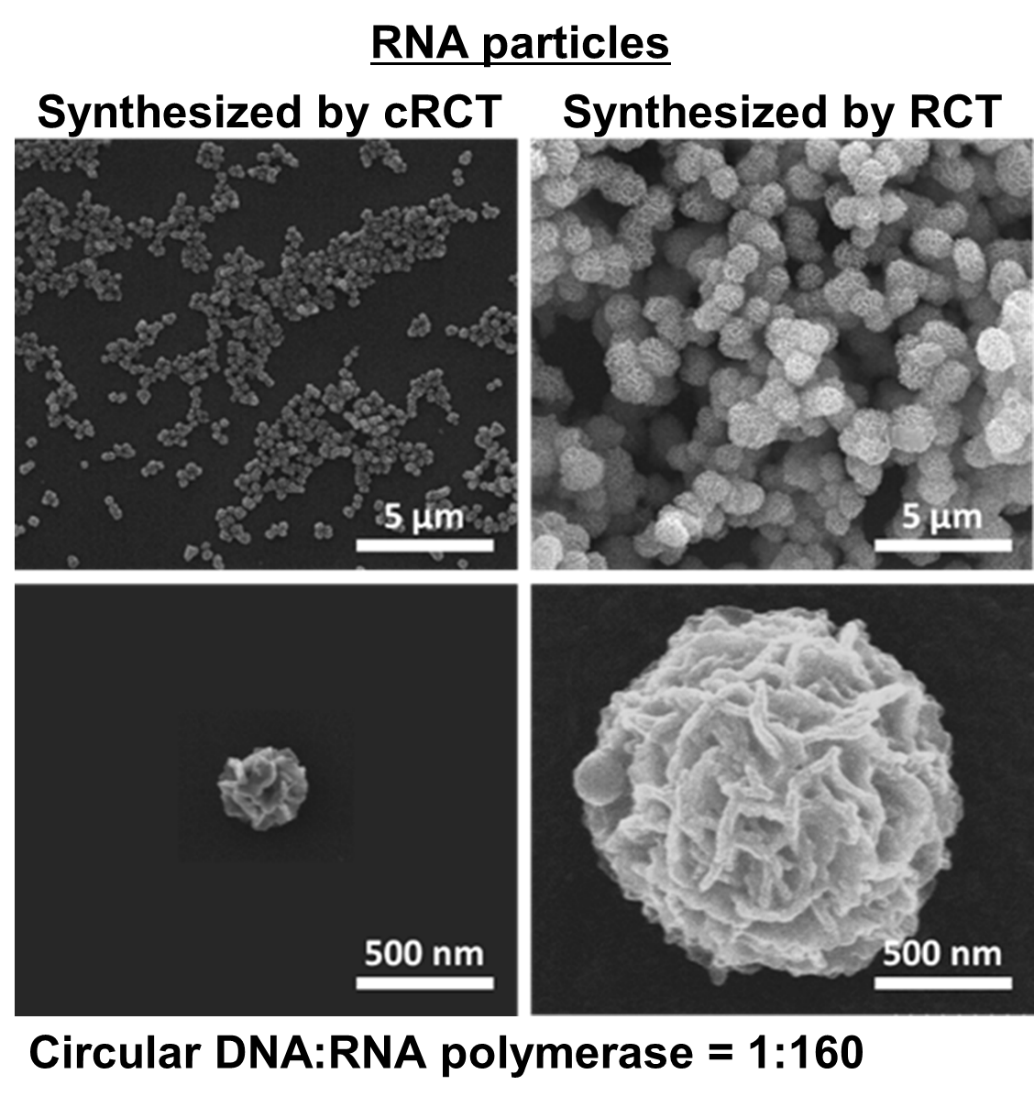


**Figure S1.** SEM images of RNA particles synthesized by complementary rolling circle transcription (cRCT) which involves two types of circular DNAs complementary to each other (left) and RNA particles synthesized by RCT with single circular DNA. Although same reaction conditions were applied for generation of both types of RNA particles, the RCT products from single circular DNA were micro-sized, while the former was about 300 nm-sized.

**Methods**

For the synthesis of RNA-NPs, circular DNA1 and circular DNA2 at the final concentration of 0.5 μM each were mixed with 8 mM of ribonucleotide solution mix, reaction buffer (80 mM Tris, 40 mM NaCl, 12 mM MgCl_2_, 4 mM spermidine, 20 mM dithiothreitol, pH 7.8) and 80 units μl^-1^ of T7 RNA polymerase. For the synthesis of RNA particles from single circular DNA, 1.0 μM of circular DNA1 was used and all the other reaction conditions were kept same as those for synthesizing BRCs. For the RCT reaction, the reaction solution was incubated for 20 h at 37°C.
